# Characteristics of HPV-unvaccinated undergraduate health students in Switzerland, a cross sectional study

**DOI:** 10.1186/s13690-019-0348-y

**Published:** 2019-06-18

**Authors:** Mona Amadane, Charlotte de Pree, Manuela Viviano, Pierre Vassilakos, Emilien Jeannot, Patrick Petignat

**Affiliations:** 1 Faculty of Medicine, University of Geneva, Rue Michel-Servet 1, Geneva, 1206 Switzerland; 2 Gynecologic Division, Department of Obstetrics and Gynecology, Geneva University Hospitals, Boulevard de la Cluse 30, 1205 Geneva, Switzerland; 3 Geneva Foundation for Medical Education and Research, Route de Ferney 150, 1211 Geneva 2, Switzerland; 4 Institute of Global Health - Faculty of Medicine, Chemin de Mines 9, 1202 Geneva, Switzerland; 5 Community Psychiatry Service, Lausanne University Hospital CHUV, Lausanne, Switzerland

**Keywords:** Cervical cancer, Human papillomavirus (HPV), Undergraduate students, Unvaccinated

## Abstract

**Background:**

Human Papillomavirus (HPV) vaccination, intended for young women aged 11–14 years old, has been introduced in Switzerland in 2007. Ten years after its introduction, only a few studies have explored the reasons associated with uptake and non-uptake of the vaccination. Our objective was to identify the sociodemographic characteristics of a population of vaccinated and unvaccinated undergraduate healthcare female students, to define the reasons of non-uptake of vaccination, and compare our findings with those found in other Swiss cantons.

**Methods:**

Between January and November 2017, women studying in Health Sciences School and Medical School in Geneva, aged 18–31 years old, were recruited in a large trial assessing HPV prevalence. As part of a smaller, observational study nested in this larger trial, women were invited to complete a questionnaire. Self-reported HPV vaccination uptake or non-uptake, as well as knowledge and attitude about HPV vaccination were assessed. T-Test and Chi square test were used to compare characteristics of vaccinated and unvaccinated women.

**Results:**

Overall, 409 women were recruited in the study. The majority of them (69.1%) reported having been vaccinated for HPV, while 30.9% of them had never received any dose of the HPV vaccine. The only factor associated with a higher vaccination rate was the participants’ origin, as women from Geneva were more represented in the vaccinated group than women from other Swiss regions or countries. Unvaccinated women were more likely to consider HPV vaccination as less important than the vaccinated ones (50.4% vs 3.5% *p < 0.001*).

**Conclusion:**

Although no typical profile can be established in this studied population of unvaccinated women, a lack of information was a major reason of non-uptake of vaccination among the study participants. An effort by health authorities and carefully designed messages are essential to increase the population’s awareness over cervical cancer and its prevention.

**Trial registration:**

The trial was registered under cliniclatrials.gov with the identifier: NCT03474211.

## Background

Human Papillomavirus (HPV) is responsible for the most prevalent sexually transmitted infection worldwide, which represents a major public health challenge [1]. It is estimated that up to 70% of the sexually active population will be infected with HPV at least once in their life [2]. The highest infection rate is found among 16–25-year-old women. While up to 70% of HPV infections are spontaneously cleared after a few months [3], the persistence of the virus is responsible for the development of cervical cancer, which is associated to HPV in nearly 100% of cases [4]. Cervical cancer is the fifth most common cancer among women aged 20–49 years living in Switzerland [5]. Every year, Switzerland counts as many as 250 cervical cancer and 5′000 cervical precancerous lesion diagnoses [6].

HPV vaccination represents a fundamental primary prevention measure for the development of cervical cancer. The currently available vaccines in Switzerland are Gardasil**®** and Cervarix**®**, both of which protect against HPV genotypes 16 and 18, which are responsible for the development of cervical cancer in over 70% of cases [7]. Gardasil**®**, which has been available on the market since 2007, also covers against genotypes 6 and 11, which are mostly responsible for the development of genital condylomas [8].

The Swiss recommendations for HPV vaccination were first published in 2007, advising the vaccination for all females between the ages of 11 and 14 years while also recommending a catch-up vaccination for women aged 15 to 19 years [9]. The aim of such primary prevention measure is to cover over 80% of the target population. Such recommendations, however, were implemented by each federal canton individually. Such individualized implementation has resulted in disparities in the campaigns and, therefore, in vaccination rates across the country (from 17 to 75% of the targeted population in 2014) [10].

In the Canton of Geneva, the first HPV vaccination campaign tookplace in September 2008. The greatest asset of the campaign in Geneva is the strong involvement of school health services, such as the *Service de L’Enfant et de la Jeunesse* (SSJ) in public schools, which inform families and offer vaccination to all female school children aged 11–19 years old. According to a study on HPV vaccination carried out 4 years after the first campaign, the majority of 13–14 year-old girls had been vaccinated through the SSJ, thus proving its efficiency [11]. Despite this institutional effort, however, the targeted coverage rate of 80% has yet to be reached. Only a few studies have been carried out with the aim of understanding the reasons for non-vaccination in Switzerland. Moreover, as the campaigns are organised individually by each canton, it is difficult to make generalisations based on the other cantons’ experience and statistics.

The aim of this study was (i) to compare the sociodemographic characteristics of a population of vaccinated and unvaccinated undergraduate female healtcare students in the Geneva canton, (ii) to define the reasons for not having undergone vaccination in the latter group, and (iii) to compare the reasons for non-vaccination to those found in other Swiss cantons.

## Material and methods

### Population

Recruitement of the study participants took place from January to November 2017 at the Medical school and at the School of Health Sciences of the Medical University of Geneva, located in the city of Geneva, Switzerland. Women aged 18–31 years, currently attending either Medical School to obtain a medical doctor degree or the School of Health Sciences in Geneva to obtain a nurse or midwife degree, were invited to participate in the study.

### Study design

This study has been carried out as a nested, observational study within a larger trial evaluating the HPV vaccine’s effectiveness by analyzing the HPV prevalence using a cervico-vaginal self-sampling method [12].

Announcements about the study were given by previously-informed professors teaching classes at the School of Health Sciences and at the Medical School in Geneva. An email was also sent by the study investigators to the students prior to their recruitement. After having delivered a short presentation about the HPV infection and the project’s design, the study investigators distributed the kits to those students who expressed an interest to participate in the study. The kits contained a cotton swab for HPV self-sampling (ClassiqSwab, COPAN, Brescia, Italy), illustrated instructions on how to use the swab for self-sampling, an informed consent form, an explanatory document about the HPV infection and a questionnaire on sociodemographics and their reasons of non vaccinated choice. Each kit also contained an identification number, to which the participants could refer to obtain their HPV test results. The students were given 1 week to return the kit and the questionnaire to the study investigators. The results of the HPV analysis were given to the students by a designated study investigator upon request.

The questionnaire which women were invited to complete in order to fulfill the aim of this study included questions about the HPV vaccination uptake or non-uptake, the participants’ knowledge and attitudes over the HPV vaccination, their sexual behavior and country or Swiss canton of origin.

### Sample size

The sample size was calculated based on the primary outcome of the main study [12]. It was obtained based on an estimated prevalence of 6% of HPV 16/18 infection in the Swiss population aged less than 30 years. A total of 400 specimens were needed to detect about an 85% reduction in HPV 16/18 prevalence (prevalence of 0.9% in the vaccinated population), given an 80% power and a two-sided significance level of 95%. We therefore estimated that a sample size of 400 women would be adequate for the analyses.

### Statistical analyses

Statistical analyses were run using STATA 13. The normality of the distribution was tested by the Kolmogorov-Smirnov test. Descriptive statistics and frequencies were analysed for all variables. The T-test and Chi square test were used for the descriptive statistics and for the comparaison between variables. A *p* value of less than 0.05 was considered as statistically significant.

### Ethical approval

The study protocol was approved by the ethical cantonal board in Geneva (Commission Cantonale d’Ethique et de la Recherche – CCER) with the identification number 15–357. All participants signed an informed consent form prior to taking part in the study. The trial was registered under cliniclatrials.gov with the identifiers: NCT03474211.

## Results

### Sociodemographic and clinical characteristics

Out of 500 kits distributed, a total of 409 were given back, thus obtaining a response rate of 81.8% (409/500). Among the 409 participants included in the study, 55% of them (225/409) were enrolled in Medical School while 45% (184/409) of them attended the School of Health Sciences. A total of 46% of the study participants reported their vaccination status based on their own personal notion, without verifying such data on their vaccination booklet. Women coming from the Geneva canton were more represented in the vaccinated (71.1%, 202/284) than in the nonvaccinated group of participants (59.2%, 74/125), whereas women coming from other Swiss cantons, who were grouped together with women coming from other countries (France, Portugal, Spain ect..) were more represented in the nonvaccinated group (40.8%, 51/125) than in the vaccinated one (28.9%, 82/284*, p = 0.017*). We found that only 2.8% (8/284) of vaccinated women were infected by the HPV strains 6, 11, 16 and 18, while up to 11% (17/125) of the unvaccinated participants were infected by these same 4 strains. The participants’ sociodemographic and clinical characteristics are reported in Table 1.

**Table 1:** Sociodemographic characteristics and HPV test results of the study population

Variable	vaccinated		unvaccinated		*p value*
	n=284		n=125		
	n	%	n	%	
Age, y					
Mean	22.5 SD (±) 2.9		21.9 SD (±) 2.6		0.16
< 20	110	38.7	50	40	**0.03** ^#^
20-23	101	35.6	57	45.6	
> 23	73	25.7	18	14.4	
Origin					**0.017**
Geneva	202	71.1	74	59.2	
Other*	82	28.9	51	40.8	
Tobacco smoking					0.31
Yes	53	18.7	27	21.6	
No	231	81.3	98	78.4	
Age at your first sexual intercourse, mean (y)	17.1 SD (±) 2.4		17 SD (±) 2.8		0.14
Total number of sexual partners					
Mean	5.3 SD (±) 1.26		5.1 SD (±) 1.33		0.42
None	10	3.5	4	3.2	0.98
≤ 5	135	47.5	60	48	
> 5	139	48.9	61	48.8	
Use of condoms					0.6
Never/sometimes	142	50	66	52.8	
Often/always	142	50	59	47.2	
HPV prevalence					
Types 6, 11, 16, 18	8	2.8	17	11	**0.0002**
Other HR and LR HPV types	41	14	25	20	0.12

*Abbreviations*: *HPV* Human Papillomavirus, *y* years, *N* number, *HR* high risk HPV, *LR* low-risk HPV

*Includes women coming either from other Swiss cantons or from other countries

^#^*p* value in boldface are statistically significant

### Beliefs regarding the importance of the HPV vaccination

Overall, 91.9% (261/284) of the vaccinated women believed that the HPV vaccination was as important as other vaccinations, while only 48.8% (61/125) of the unvaccinated participants believed that the HPV vaccination was as important as the others *(p < 0.001*). A total of 4.6% (13/284) and 0.8% (1/125) vaccinated and unvaccinated women, respectively, believed that the HPV vaccination was more important than other types of vaccination. There were 3.5% (10/284) and 50.4% (63/125) of vaccinated and unvaccinated women, respectively, who believed that the HPV vaccination was less important than others. The participants’ perceptions of the importance of the HPV vaccination are reported in Table 2.

**Table 2:** Participants’ beliefs about the HPV vaccination

In general, do you think that HPV vaccination is a vaccination:	Vaccinated		Unvaccinated		*P value*
	N	%	N	%	
More important than others	13	4.60	1	0.80	**< 0.001** ^#^
Less important than others	10	3.50	63	50.40	**< 0.001**
As important as the others	261	91.90	61	48.80	**< 0.001**

*Abbreviations*: *HPV* Human Papillomavirus, *N* number

^#^*p* value in boldface are statistically significant

### Association between opinion on the HPV vaccination and sociodemographics characteristics

Among unvaccinated participants, the proportion of women who believed that the HPV vaccination was less important than others decreased as the women’s age increased (62% of the < 20 years group (31/50), 45.6% of the 20–23 years (26/57), 38.9% of the > 23 years (7/18); *p = 0.35*). On the contrary, the proportion of women who believed that the HPV vaccination was either more than or as important as other vaccinations increased with the women’s age (< 20 years: 38% (19/50); 20–23 years: 54.4% (31/57); > 23 years: 61.1% (11/18); *p = 0.07*).

### Association between opinion about the HPV vaccination and condom use

**Fig. 1:**
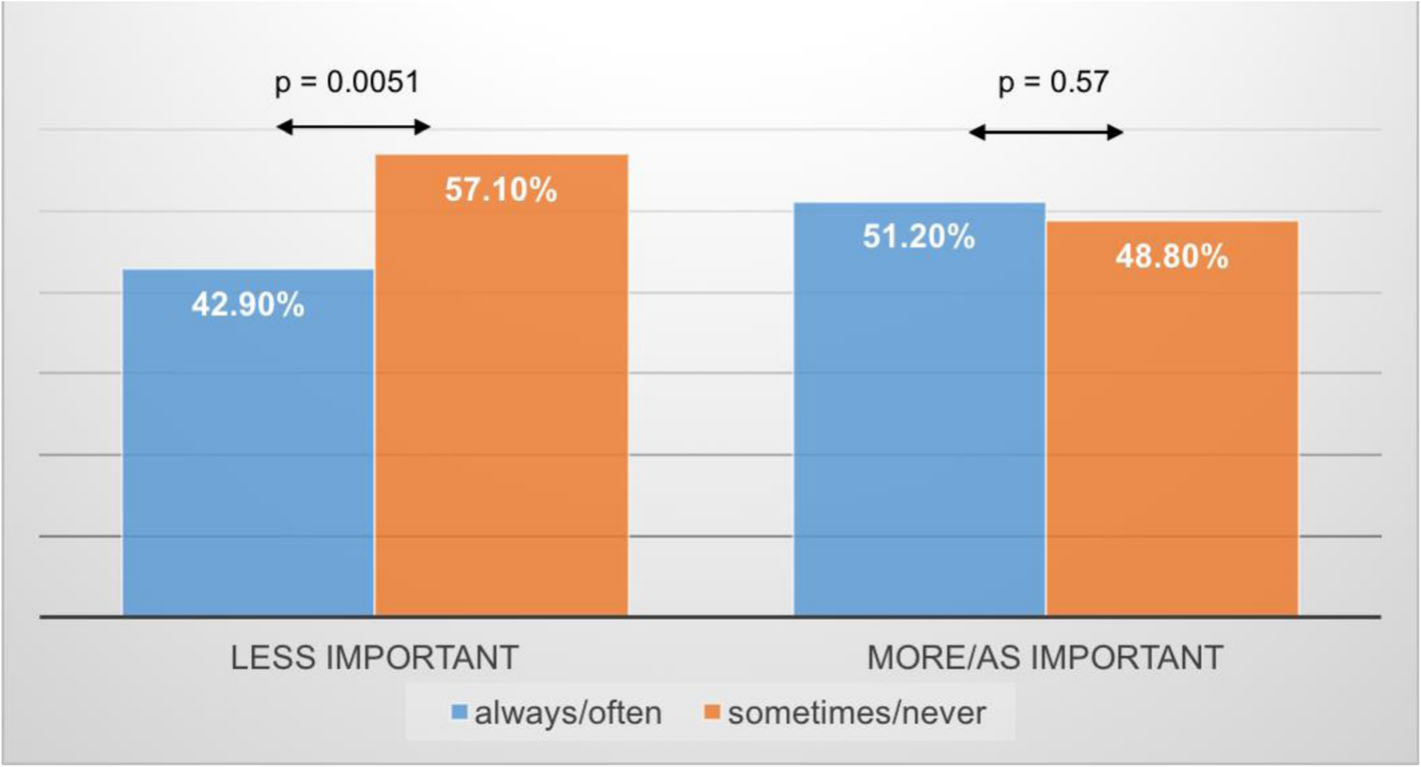
Association between use of condom and opinion about HPV-vaccination among the unvaccinated group of participants

Among unvaccinated participants who believed that the HPV vaccination was less important (50.4%; 63/125), 57.1% of them (36/63) used the condom sometimes/never, whereas 42.9% (27/63) of them used it often/always. Among women who considered the HPV vaccination as/more important than other vaccinations (49.6%; 62/125), 51.2% (32/62) used the condom often or always, while 48.8% (30/62) used it sometimes/never. The association between the participants’ opinion about the HPV vaccination and their frequency of condom use is reported in Fig. 1.

### Reasons for not having been vaccinated

A total of 41.6% (52/125) of the unvaccinated women did not give any information or did not know why they had not been vaccinated. Among women who gave a reason for not having been vaccinated (58.4%, 73/125), the reported reasons included: fear of side effects (21.6%, 27/125); parents being against the vaccination, either in general or the HPV-one (14.4%; 18/125); the physician in private practice being against the HPV vaccination (8.8%; 11/125); the vaccination not being considered as useful (2.4%; 3/125); the person being against vaccinations in general (2.4%; 3/125); sexual inactivity (1.6%; 2/125); insufficient evidence on the vaccine’s efficacy and side effects (1.6%; 2/125). The reasons for not having been vaccinated are reported in Table 3.

**Table 3:** Reasons for not having been vaccinated among unvaccinated women

Variable	n	%
No reason given or doesn’t know	52	41.6
Reason given :	73	58.4
Fear of side effects	27	21.6
Parent against HPV vaccination	18	14.4
Physician in private practice against HPV vaccination	11	8.8
Not considered useful	3	2.4
Against vaccinations in general	3	2.4
No sexual activity	2	1.6
Only 1 sexual partner	2	1.6
No efficacy	2	1.6
Insufficient evidence	2	1.6
Other	3	2.4

*Abbreviations*: *N* number, *HPV* Human Papillomavirus

## Discussion

This is the first study to evaluate the acceptability of the HPV vaccine in Geneva since the introduction of the HPV vaccination in the canton. Our results revealed that 69.4% of our study population was vaccinated against HPV, a rate higher than the rest of Switzerland where, according to the results of a survey conducted in 2016, only 53.6% of women aged 18–24 years were vaccinated [13]. Another study conducted in 2014 found that the French-speaking Swiss regions vaunt a vaccination rate of 68.1%, which is consistent with the rate found in our trial [10]. Moreover, a recent systematic review collecting data from 28 countries pointed out the heterogeneity of vaccination rates worlwide, varing from 2.4 to 94.4% [14].

However, Our results reflect a reality in which, despite the remarkable efforts to reach the optimal vaccination coverage rate, the resistance to the vaccination prevents public health workers from reaching the optimal coverage rate. When looking at reasons for non-vaccination, three of them stood out: fear of side effects (21.6%), parents being against the HPV vaccination (14.4%) and the physician being against the vaccination (8.8%). Similarly, in a study including women aged 18–24 years living in the French-speaking region in Switzerland [10], the main reasons for not having been vaccinated were: thinking it was too late (due to either age, sexual activity, or pathological smear) (52%), fear of side effects (26%), not having received enough information (19%), being against all kinds of vaccination (17%) and having discouraging relatives and friends (15%). Furthermore, a study including 16–20 year-olds living in the Canton of Vaud [9] revealed a lack of information about the HPV infection’s natural history and prevention, as over 70% of the interviewed population felt insufficiently informed about the disease. Moreover, one of the issues highlighted by 29% of the physicians working in private practice in the French-speaking region of Switzerland [8] was the lack of information and support brought by their cantons. A recent study conducted in Switzerland following the first 2 years after the vaccination’s introduction reported that only 117 cases of Gardasil®-associated side effects were found among 420′000 vaccine doses. The same study estimated that 93–98% of CIN2+ lesions caused by 16 and 18 genotypes and 46–70% of the CIN2+ lesions caused by other HPV genotypes could be avoided with vaccination [15]. Such results, which can be used by public health workers to improve the future vaccination campaigns, demonstrate that the lack of information about the HPV infection and its prevention concerns not only the general population, but also health professionals.

Concerning the opinion about the importance of the HPV vaccination, we found that the difference between vaccinated and unvaccinated women was compatible with their immunization status. Over 50.4% of unvaccinated women considered the vaccination as less important than other vaccinations, while 91.9% of vaccinated women considered it as as important as other vaccinations. Another study carried out in Italy obtained similar results among women aged 18–21 years old, thus demonstrating that one of the factors associated with not having been vaccinated was the lower perception of its benefits [16].

A small majority (51.1%) of our population sample had been vaccinated between the ages of 15 and 19 years old, although vaccination for women in this age range was meant to be a catch-up for those who had missed their opportunity to be vaccinated in the first place. Knowing that the moment for the ideal vaccination is before first sexual intercourse, which in our population took place at a median age of 17.2 years old, baseline vaccination for all 15–19 years-olds seems to be a reasonable target to improve prevention. Nevertheless, the median age when receiving the first vaccine dose in our study population was 14.8 years old. The small percentage of vaccinated women after the age of 20 years old (4.2%) is not surprising considering that the campaign mainly targets the younger part of the population. Such results are in line with those of a study [6] evaluating the age at the first dose of HPV vaccine in a population of Swiss women, which found that the vaccination rates were 54.4% for women between aged 15–19 years old, 39.8% for women aged 11–14 years old, and 5.8% for women older than 20.

When studying the sociodemographic characteristics of our population, only nationality was found to be significantly associed to vaccination status, as a greater proportion of women coming from Geneva and its surroundings were vaccinated when compared to women coming from other cantons and countries. Given the difference of the vaccination campaigns and policies in other cantons and countries, such finding highlights the efficacy of the vaccination campaign in the canton of Geneva, where a particularly active role was played by the SSJ in public and private schools. Other studies have confirmed that, when the SSJ was involved in vaccination campaigns, as is the case in other French-speaking cantons in Switzerland, such finding resulted in better vaccination rates than those in the German-speaking part of Switzerland [9].

One strength of our study was given by the fact that we chose a population of young, future healthcare providers, whose opinion is fundamental in the view of spreading the vaccination uptakein the near future. In addition, this population sample of young adults has never been studied in such geographical area.

One limitation of our study was the sample’s relatively small size, which limited the power of some of our observations. A selection bias also may have occurred, as all the participants not only had a high educational degree but also studied medical and health sciences, which does not reflect the heterogeneity of the general population. Additionally, the HPV kits were offered to the students who proactively expressed an interest to participate in the study, excluding the girls possibly having another opinion about the HPV vaccination. Finally, 46.3% of our participants had not checked their vaccination record to answer the questionnaire, an aspect which may have altered some of the study results.

## Conclusion

The suboptimal HPV vaccination rate among our study population of undergraduate women shows that, despite the vaccine’s proven efficacy, the coverage rate is still far from reaching 80%. The majority of vaccinated women in our study population came from the Geneva Canton, a finding which further highlights the discrepancies in vaccination campaigns in the country. Proactive education about the HPV infection’s natural history and the vaccination’s role, to be delievered by the women’s personal healthcare providers, represents a fundamental step in increasing the vaccination coverage rate across the country.

## Abbreviations

CCER: Commission Cantonale d’Ethique et de la Recherche
HPV: Human PapillomaVirus
SSJ: Service de L’Enfant et de la Jeunesse
